# Implementing effective salt reduction programs and policies in low- and middle-income countries: learning from retrospective policy analysis in Argentina, Mongolia, South Africa and Vietnam

**DOI:** 10.1017/S136898002100344X

**Published:** 2022-03

**Authors:** Jacqui Webster, Joseph Alvin Santos, Martyna Hogendorf, Kathy Trieu, Emalie Rosewarne, Briar McKenzie, Lorena Allemandi, Batsaikhan Enkhtungalag, Ha Thi Phuong Do, Pamela Naidoo, Clare Farrand, Temo Waqanivalu, Laura Cobb, Kent Buse, Rebecca Dodd

**Affiliations:** 1 The George Institute for Global Health, University of New South Wales, PO Box M201, Missenden Rd, Newtown, NSW 2050, Australia; 2 Independent Nutrition Consultant, Geneva, Switzerland; 3 Independent Nutrition Consultant, Washington, DC, USA; 4 Public Health Institute, Ulaanbaatar, Mongolia; 5 National Institute of Nutrition, Hanoi, Vietnam; 6 Heart Foundation, Cape Town, South Africa; 7 World Health Organization, Geneva, Switzerland; 8 Vital Strategies, New York, USA

**Keywords:** Salt reduction, Low- and middle-income countries, Health policy analysis, Non-communicable diseases

## Abstract

**Objective:**

To understand the factors influencing the implementation of salt reduction interventions in low- and middle-income countries (LMIC).

**Design:**

Retrospective policy analysis based on desk reviews of existing reports and semi-structured stakeholder interviews in four countries, using Walt and Gilson’s ‘Health Policy Triangle’ to assess the role of context, content, process and actors on the implementation of salt policy.

**Setting:**

Argentina, Mongolia, South Africa and Vietnam.

**Participants:**

Representatives from government, non-government, health, research and food industry organisations with the potential to influence salt reduction programmes.

**Results:**

Global targets and regional consultations were viewed as important drivers of salt reduction interventions in Mongolia and Vietnam in contrast to local research and advocacy, and support from international experts, in Argentina and South Africa. All countries had population-level targets and written strategies with multiple interventions to reduce salt consumption. Engaging industry to reduce salt in foods was a priority in all countries: Mongolia and Vietnam were establishing voluntary programs, while Argentina and South Africa opted for legislation on salt levels in foods. Ministries of Health, the WHO and researchers were identified as critical players in all countries. Lack of funding and technical capacity/support, absence of reliable local data and changes in leadership were identified as barriers to effective implementation. No country had a comprehensive approach to surveillance or regulation for labelling, and mixed views were expressed about the potential benefits of low sodium salts.

**Conclusions:**

Effective scale-up of salt reduction programs in LMIC requires: (1) reliable local data about the main sources of salt; (2) collaborative multi-sectoral implementation; (3) stronger government leadership and regulatory processes and (4) adequate resources for implementation and monitoring.

Excess salt intake causes high blood pressure^(1,2)^, which increases CVD risk^(3,4)^. About two million deaths per year globally can be attributed to people eating more than the WHO’s recommended daily salt intake of less than 5 g/d^(5)^. People in most countries consume more than this target, and the global mean salt intake is estimated to be about 10 g/d^(6)^. Whilst the mean intake tends to be lower for low- and lower-middle-income countries (LMIC)^(7)^, the range is wide, with some regions particularly East Asia and Central and Eastern Europe, having very high salt intakes^(6–8)^.

Many studies show that salt reduction is a cost-effective or even cost-saving approach to lower blood pressure and reduce risk of CVDs^(3,9,10)^. It is therefore considered a priority action for non-communicable disease (NCD) prevention^(11)^. In 2013, WHO recommended that Member States establish programs to reduce salt intake by 30 %, as part of efforts to reduce the burden of NCD by 25 % by 2025^(12)^. The benefits of implementing salt reduction programs were further emphasized with its inclusion in the WHO Best Buys^(13)^. Repeated systematic reviews have shown an increase in the number of countries with national salt reduction strategies from 32 in 2010^(14)^ to 75 countries in 2014^(15)^, to 96 in 2019^(16)^. A Cochrane systematic review summarised the available findings on impact and demonstrated intervention effectiveness in some countries^(17)^. To date, few upper-middle-income countries have reported a reduction in population salt intake, and no low or lower-middle-income countries have achieved a reduction^(16,18)^.

Given that more than 85 % of premature deaths worldwide attributable to NCD are in LMIC^(19)^, the objective of this review is to understand factors that influence the implementation of salt reduction programs with a view to improving impact in these settings.

## Methods

The study is a retrospective policy analysis based on desk reviews and interviews from four LMIC in 2018.

### Countries

Countries were purposefully selected (Argentina, Mongolia, South Africa and Vietnam) to provide a range of regions and experiences on implementation of salt reduction interventions. Argentina is an upper-middle-income country with more than 40 million people. It has a large rural population and food processing is one of its leading industries, making it an important case study for salt reduction. Mongolia is a landlocked country in East Asia: 45 % of the 3·3 million population live in the capital city Ulaanbaatar, about 30 % are nomadic. It has little agricultural land for growing food so it relies mainly on livestock farming and mining as key sources of income. These three factors provide unique challenges for dietary interventions. South Africa is classified by the World Bank as a newly industrialised country^(20)^. With almost 60 million people, it is a multi-ethnic society with a variety of cultures, religions and languages. Despite a well-organised food industry, it was the first country to establish legislation to limit salt levels in foods. Vietnam is a socialist country in South East Asia with over 96 million inhabitants. Since economic and political reforms, commencing in 1986, it has a growing Gross Domestic Product and relatively advanced public health infrastructure. All countries started developing salt reduction programs around 2009/2010 but have progressed at different rates.

### Desk reviews

Relevant literature on each country between 2010 and 2018 was extracted from an existing database of salt reduction interventions^(15)^, and supplemented through a search of published and grey literature. Information from the desk review was used to develop detailed country overviews (or case studies) of salt strategies based on the WHO SHAKE package (see below), which informed the stakeholder interviews.

### Stakeholder interviews

Eight to fifteen people from each country were invited to participate in stakeholder interviews between July and December 2018. Existing government contacts identified key people from relevant sectors, including government and non-government health organisations, research institutes, food industry, and institutions serving food. Participants were involved in salt reduction strategies in varying capacities, including policy work, research, implementation, evaluation and monitoring. In-depth semi-structured interviews were conducted either in-person or through teleconference (South Africa only). Participants were provided with information on the purpose of the study, informed that the interview would be recorded, and asked to sign a consent form with the option to withdraw at any point. Interviews lasted from 30 to 60 min. Where interviews were conducted in the local language, simultaneous interpretation was provided by a local translator. All interviews were audio-recorded and transcribed by an external provider. Questions varied between stakeholder groups and individual participants but followed a guiding document distributed to participants prior to the interview.

### Analytical framework

The areas of inquiry were informed by the Walt and Gibson’s Health Policy Triangle^(21)^. This framework helps to systematically identify and assess factors that may affect policy. Sides of the triangle represent domains of analysis: context, process and content. For our analysis, we understood context to cover barriers and enablers to policy implementation including political environment and availability of local data; process to cover agenda setting, policy formulation and implementation and evaluation and content to cover the aims of relevant policies and the intended activities. Key actors were also identified and ‘sit’ in the centre of the triangle. We also asked interview participants to identify lessons that might be applicable in other countries.

### Data analysis

The analytical framework was used to guide data extraction and analysis. Relevant information on context, process, content and actors was extracted from the country case studies and the interview. The WHO SHAKE^(22)^ package was used as a benchmark against which to assess content according to the main domains: **S**urveillance (data collection and monitoring), **H**arnessing industry (including setting reformulation targets and use of salt substitutes), **A**ction on labelling and marketing, increasing **K**nowledge and strategies to change the **E**nvironment (including activities implemented in settings such as hospitals, schools, workplaces and catering facilities). Two research members independently applied codes to each transcript and discussed coding nuances to achieve consensus and strengthen inter-coder reliability. Data coding was completed using NVivo 12. Analysis was undertaken to assess commonalities, differences and recurring themes.

## Results

**Fig. 1:**
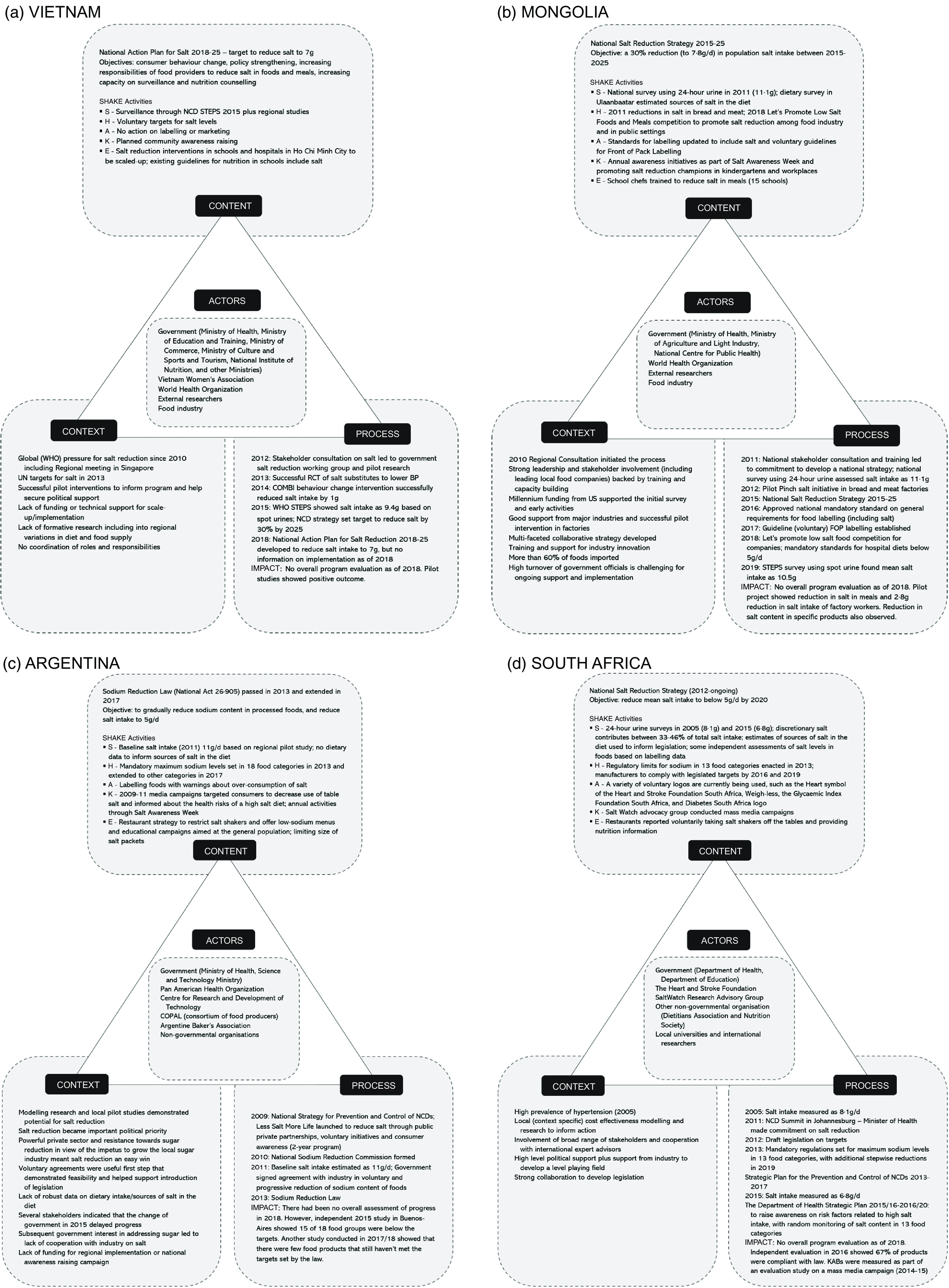
Analysis of National Salt Reduction Policies using Walt and Gibson’s Health Policy Triangle: (a) Vietnam; (b) Mongolia; (c) Argentina; (d) South Africa

Informant interviews were undertaken in Argentina (7), Mongolia (13), South Africa (4) and Vietnam (11). Figure 1 demonstrates the analysis based on the Health Policy Triangle to outline context, process (agenda setting/policy formulation, policy implementation, evaluation), content and actors engaged in the salt reduction programs for each country. Table 1 drills into ‘context’ and presents more details on the barriers and opportunities to implementation.

**Table 1:** Understanding the context (barriers and enablers) impacting implementation of salt reduction strategies in relation to different elements of the SHAKE package based on stakeholder interviews in four countries

SHAKE framework	Argentina		Mongolia		Vietnam		South Africa	
	Barriers	Enablers	Barriers	Enablers	Barriers	Enablers	Barriers	Enablers
S—Surveillance (Measuring and monitoring)	• Lack of recent dietary intake data and reliance on industry information	• Strong technical expertise in monitoring • Fed government commitment to monitoring • Involvement of provincial authorities • External funding	• High lab costs for analysis • Lack of access to laboratory equipment • No data on nutritional composition of meals	• Baseline data required to initiative work to implement strategy • External funding	• No nationally representative 24-h urine survey • WHO STEPS survey only used spot urines	• Individual knowledge of individual salt intake would likely stimulate changes in behaviour	• Lack of 24-h urine data • Capacity to robustly measure salt levels in foods (compliance with legislation)	• Development of standard testing procedures for labs
H—Harnessing industry to reformulate	• Many small producers with limited technical knowledge in key categories (e.g. bread) • Consumer acceptability • No support for salt substitutes due to perception that need to change consumer taste perceptions	• High market concentration in some categories (e.g. soups) • Research into acceptability • Technical support from government	• Outdated food standards	• Sharing case studies on best practice • Competition on innovation	• Limited technical capacity/need for technical guidance • Lack of consumer demand for reduced salt products • Lack of manufacturer capacity to scale-up salt substitutes • Lack of government support for salt substitutes	• National Plan includes salt targets and highlights industry responsibility • Opportunity to scale-up salt substitutes based on existing small-scale production	• Two-stage targets mean need to update labels twice • Large informal food sector (unregulated) • Government perceives increased cost of salt substitutes is a barrier to market introduction	• Industry support for regulation • Success story with bread—early movers • Competition to achieve the targets
A—Action on Standards for labelling and marketing	• Lack of agreement on FOPL • No enforcement	• None identified	• Manufacturers bulk-buy labels • No consistent labelling for restaurants and caterers • No regulatory measures	None identified	• No skills or capacity to market reformulated products	None identified	• Lack of enforcement • People don’t know how to read labels	• Good labelling policy
K—Knowledge (Education and communication strategies)	• No public awareness • Lack of funds or capacity for communication campaigns	• World Salt Awareness Week	• Initial level of knowledge and behaviours very low • No strategic program or theoretical framework for behaviour change • Hard to sustain over time	• World Salt Awareness Week • Training and use of media	• Vietnamese traditionally consume a lot of salt • Regional differences	• Successful pilot study (although not generalisable • Most of salt is added during cooking or at the table	• Mass communication is expensive • Lack of dedicated ongoing funding	• Increased recognition for public health • Growing movement with increasing opportunities to communicate messages • One off injection of government funds • Use of well-known comedian as salt champion
E—Environment-supporting settings for healthy eating	• Restaurants only attended by middle classes • No policies for schools	• Regulation to take saltshakers off the table in restaurants	None identified	• Opportunity to both train chefs and educate students and employees • Promotion of champions • Development of guidelines	• The National Action Plan on Salt only includes interventions in schools and workplaces (not hospitals or other public institutions	• Successful pilot study in the workplace (factories)	• No regulation on taking saltshakers off the table	• Guidelines for schools and hospitals • Education and training for government workplaces • Nutrition information provided by restaurants/fast food venues • Nutrition education part of school curriculum

### Context

#### Drivers behind salt strategies

A mixture of national and international drivers influenced the development of salt reduction strategies. Informants from Mongolia and Vietnam cited a WHO Western Pacific Regional consultation meeting in Singapore in 2010, whereas Argentina and South Africa pointed to high levels of hypertension, locally available context-specific research and collaboration with international experts, as main drivers. Lack of formative research, including understanding regional variations in diet and food supply and lack of funding for scale-up, was identified as barriers to establish the salt reduction programs in Argentina and Vietnam. Argentina, Mongolia and South Africa highlighted strong government leadership and stakeholder involvement as facilitators. Mongolia additionally highlighted funding from the US Millennium Challenge Fund (2012), as influential in driving change. In Argentina, robust industry resistance to any action to reduce sugar consumption was also thought to have resulted in the food industry being more inclined to act on salt reduction to placate and maintain good relations with the government. All countries additionally identified the 2011 UN high-level meeting on NCD^(23)^ as influencing their decisions to act on salt reduction.

### Process

#### Agenda setting/policy formulation

Mongolia and Vietnam had similar processes for establishing their salt reduction strategies. Both programs were informed by national stakeholder consultations in 2011–2012, in collaboration with WHO and supported by international experts. Local research and successful pilot studies also supported program implementation—for example in Mongolia, a pilot project demonstrated a 2·8 g reduction in salt intake of factory workers^(24)^. Mongolia’s approach benefitted from a substantial injection of foreign funds, which enabled robust local data collection. Argentina and South Africa took different approaches to establishing legislation to limit the salt content in certain foods. Argentina first established voluntary initiatives to engage the food industry in food reformulation and a consumer awareness campaign (2-year program) in 2009, as a precursor to the Sodium Reduction Law in 2013^(25)^. In contrast, after many years of local research and advocacy which commenced in 2005, South Africa committed to salt reduction in 2011 and consulted on draft legislation for salt content targets for foods in 2012^(26)^. This led to the introduction of mandated maximum sodium levels established in 2013 to be achieved by 2016, with additional reductions to be achieved by 2019^(27)^. Government commitment to salt reduction was further outlined in the NCD Plan 2013–2017, to be implemented through the Department of Health 2016–2020^(28)^. Three of the four countries now have national government action plans to reduce salt. Argentina’s commitment to salt reduction is incorporated into the Healthy Argentina Plan as part of the National Strategy for the Prevention and Control of NCD.

#### Policy implementation

All countries highlighted similar challenges and opportunities relating to implementation. Whilst legislation was introduced in Argentina and South Africa, changes in leadership and lack of funds for monitoring and evaluation hampered effective implementation in both countries. In Argentina, implementation of national laws by regional jurisdictions was patchy, and the National Institute of Food Products in charge of monitoring the law only conducted chemical analysis in some provinces. Strong collaboration and political support were cited as enablers to start with the voluntary initiative in Argentina, Mongolia and South Africa compared to Vietnam where, despite leadership and commitment from Ministry of Health, stakeholders identified the lack of coordination as a barrier. Voluntary agreements with the food industry to reduce salt in foods, early food industry successes in reformulating foods to contain less salt (particularly bread) and training and support for food industry innovation were highlighted as key facilitators in Argentina and Mongolia.

#### Evaluation

Evaluations suggested none of the countries had reduced salt intake by 2018 when this research took place. Argentina, Mongolia and South Africa reported reducing salt levels in some processed foods. Evaluation conducted in South Africa in 2016 showed 67 % of products were compliant with the first round of legislated targets for reduced salt levels^(29)^. Sub-national studies conducted in Argentina showed most food groups were below the salt targets set by the law and/or the regional targets for Latin America^(30,31)^. In Mongolia, companies had reported reductions in bread products and selected processed meats^(24)^. In relation to changes in knowledge, attitudes and behaviour, an evaluation study on a mass media campaign in South Africa (2014–2015) showed significant improvements of reported behaviours to reduce salt, particularly adding salt during cooking and at the table, following the intervention^(32)^. However, interviewees stated there was no ongoing process to ensure that these changes could be sustained over time. Overall, none of the countries had a comprehensive ongoing evaluation program.

### Content

#### Existence of a national plan or strategy and population targets

Most countries had strategies and action plans to reduce salt. Mongolia (2015–2025)^(33)^, South Africa (2012 onwards)^(34)^ and Vietnam (2018–2025)^(35)^ had existing strategies and/or action plans including population targets to reduce salt. Argentina had a law establishing a limit on the sodium content of certain foods^(36)^ and the broader salt reduction program was outlined as part of the NCD Action Plan (2009)^(37)^. Salt intake targets ranged from population averages of <5 g/person per d in South Africa and Argentina, to 7 g/d in Vietnam (as well as a 30 % reduction), and to 7·8 g/d in Mongolia.

##### Surveillance

No country had a comprehensive surveillance program, and approaches to measuring salt intake were sub-optimal i.e. they were unable to evaluate change over time (Table 2). Both Argentina and Mongolia highlighted funding and technical capacity as vital for measuring salt intake. Interviewees in South Africa reported that having to rely on spot urine samples to estimate population salt intake rather than having accurate nationally representative data using the recommended 24-h urine method was a barrier to effective action, as it meant it would be difficult to demonstrate change and harder to convince policy makers to act. No country had a comprehensive assessment of sources of salt in the diet which is essential to inform interventions. Argentina’s laws were based on a dietary survey from 2004 to 2005, and targets were set according to information provided by the industry. The other three countries relied on regional studies. Only South Africa had an established process for monitoring salt levels in processed foods through labels, but still lacked capacity to assess compliance of labelling through laboratory analysis. Argentina relied on independent researchers for monitoring compliance with the law as well as the National Institute of Food Products for Chemical Analysis. Likewise, in Mongolia, prohibitive laboratory costs for analysing the sodium content in foods and absence of data on nutritional composition of meals were viewed as barriers to effective surveillance to support industry salt reduction claims. Mongolia, South Africa and Vietnam had also collected some data on knowledge, attitudes and behaviour related to salt, but there were no ongoing programs.

**Table 2:** Surveillance of salt intake, sources of salt in the diet, salt levels in foods and knowledge, attitudes and behaviours related to salt from the four countries

Country	Surveillance data			
	Salt intake	Sources of salt	Salt levels in foods	KAB
Argentina	Regional survey (24 h urine) 11 g (2011)^(38)^	2004–2005 dietary survey identified main contributors to salt as breads (20·3 %), meat products (18·7 %), cheeses (15 %), bouillon cubes or powder (11·5 %), puff pastry (9·2 %) and instant soups (6 %)^(39)^	Independent research on salt levels in foods^(30,31,40)^	Frequency of discretionary salt use (2013–2014)^(41)^
Mongolia	National survey (24 h urine) 11·1 g (2011)^(24)^ NCD STEPS (spot urine) 10·5 g (2019) (unpublished)	Study in Ulaanbaatar showed salty tea, sausages, processed meats and bread as the main sources of salt in the diet^(24)^	None available	WHO STEPS survey^(24)^
South Africa	National survey (24 h urine) Median: 6·8 g (2015)^(42)^	Regional study identified bread (25 %), beef sausages, steak and kidney pie, soup powder and margarine as the top five sources^(26)^	Vitality Institute FoodSwitch database enables regular monitoring^(29)^	Targeted research^(43)^
Vietnam	NCD STEPS survey (spot urine) 9·4 g (2015)^(44)^	Regional study (Hanoi) showed salt, salty condiments and fish sauce contributed to around 75 % of sodium in the diet^(45)^	None available	WHO STEPS survey^(44)^

KAB, Knowledge, attitudes and behaviour; STEPS, STEPwise approach to surveillance.

##### Harnessing industry

In all countries, national strategies, plans or programs highlighted the responsibilities of food industry to reduce salt but approaches were different. Vietnam planned to obtain voluntary commitments from industry with a view to setting targets for processed foods. However, the need for technical guidance was highlighted as a major barrier. Mongolia secured reductions in salt in bread and bakery products following discussions with major companies in 2011. This helped motivate further industry action but the high volume (60 %) of imported goods was identified as a barrier to effective domestic industry action. Stakeholders in South Africa pointed to industry support for regulation (to create a level playing field) and strong government leadership as major facilitators behind the establishment of legislation^(34)^. However, stakeholders highlighted the need to update labels twice (when food composition changed to meet new targets), and the large informal food sector, as barriers to implementation. In comparison, Argentina first introduced voluntary targets in 2011, then passed mandatory targets for selected processed foods through the Sodium Law in 2013^(37)^. Stakeholders highlighted the high market concentration in certain products (e.g. soups) and technical support from government as factors facilitating implementation of the law. Conversely, the high proportion of products made by small producers (with limited knowledge/technical capacity on how to reduce salt content) and consumer resistance to reduced salt foods were viewed as major barriers to implementation in Argentina. In Argentina, Mongolia and South Africa, industry competition and innovation were also seen as positive enablers for change. There were mixed views from different stakeholders about benefits of low sodium salts in all countries. While governments acknowledged the potential, other stakeholders identified lack of government support and food companies highlighted consumer acceptability and local production capacity as barriers to implementation.

##### Action on labelling

Different approaches to improving labelling to support salt reduction were identified. Mongolia had updated its nutrition labelling standards to include sodium and was introducing voluntary guidelines for Front of Pack Labelling. Vietnam was also planning to update its labelling decree and considering Front of Pack Labelling. Argentina had included high salt warnings for meals in restaurants in the 2013 Sodium Law^(37)^, but interviewees indicated that this had not been implemented. In South Africa, a variety of non-governmental organisation voluntary logos were used. Substantial barriers identified included lack of agreement on best approach (Argentina), no capacity for enforcement (Argentina/South Africa), absence of consistent standards or regulatory measures and bulk buying of labels (which is a disincentive to changing labels (Mongolia)), and lack of skills or capacity to market reformulated products (Vietnam).

##### Knowledge

No country had a coordinated strategic approach to change behaviour. Vietnam was planning national awareness raising activities as well as scaling up pilot projects implemented in schools and hospitals to other regions. Stakeholders in Vietnam highlighted the successful pilot programs to change behaviour and the fact that most dietary salt is added during cooking and at the table as facilitators to effective behaviour change. However, regional differences and the fact that salt was part of the traditional diet in Vietnam were identified as barriers. Argentina and Mongolia both undertook annual awareness raising activities as part of World Salt Awareness Week and highlighted the media, training and support materials, as important behaviour change enablers. Mongolia also employed salt reduction champions in kindergartens and workplaces. Argentina had one campaign (2011) to inform consumers about the health risks of a high salt diet and encourage less use of table salt, but this ceased due to lack of funding. Lack of dedicated funding was also identified as a barrier in South Africa, while Mongolia highlighted the lack of a strategic program or theoretical framework and challenges of sustaining activities over time as barriers to effective behaviour change programs.

##### Environment

No country had a coordinated program across schools, hospitals and workplaces but all had guidelines for different institutional settings. In Mongolia, school chefs were trained to reduce salt in meals in 15 schools as part of a pilot project and there were plans at the time to extend this to other regions. Mongolia highlighted the opportunity to both train chefs and educate students and employees and the promotion of salt champions as key enablers for implementation of the salt reduction strategy in schools and hospitals. Vietnam pointed to the successful pilot intervention in factories as a potential enabler for other workplace interventions (such as government offices or other factories). South Africa highlighted the availability of education and training for government workplaces, nutrition information provided by restaurants/fast food venues and the fact that nutrition education was part of school curriculum as key enablers for salt reduction interventions, even though these programs were not salt-specific. Argentina’s Sodium Law was extended to restaurants in 2017 and supplemented with a restaurant-specific strategy. Together these initiatives led to the removal of saltshakers on tables, limits on the size of salt sachets and introduction of low-sodium menus and mandating high salt warnings in restaurants. However, stakeholders noted that only the upper and middle classes go to restaurants so the policy of banning saltshakers was not targeting everyone and there was no monitoring.

### Actors

Governments (federal and provincial), the WHO and researchers were identified as key actors, and there had been extensive stakeholder consultation, including with a range of Ministries, to establish salt reduction plans in the four countries. In Mongolia and Vietnam, WHO had collaborated with Ministry of Health on initial consultation and training meetings, supported by international experts and continued to provide technical support in both countries. The Pan American Health Organization played an important role in Argentina, and international experts were also key influencers in Argentina and South Africa. Food companies were identified as powerful actors in Argentina and Mongolia. Salt champions—particularly school chefs—were highlighted as key actors in Mongolia, and the non-governmental organisation (South African Heart and Stroke Foundation) was considered critical to program implementation in South Africa. Strong political leaders were cited as advocates for change and key enablers for the programs in Argentina and South Africa. Additionally, both civil society groups and professional associations lobbied the government to act on salt in South Africa. Conversely, changes in government or changes in personnel in key roles were identified as a barrier to effective implementation in all countries.

## Discussion

This study of the factors influencing the design and implementation of salt reduction programs in four countries provides important insights into the challenges faced and provides guidance to strengthen future programs. Walt and Gilson’s ‘framework^(21)^ enabled us to more systematically identify and understand the roles of key actors, program content, the processes of agenda setting and policy implementation and the context influencing the initiatives across the countries. This helped to facilitate cross-country comparisons and identify common themes and lessons learned that are relevant for implementing the WHO SHAKE interventions in different contexts.

Whilst all four countries started considering salt reduction around 2010, they identified different drivers. WHO targets, and the Western Pacific Regional consultation on salt reduction in Singapore, were identified as catalysts for action in Mongolia and Vietnam. This highlights the importance of global strategies to motivate country action. Catalytic funding for pilot studies helped secure the local evidence to foster demand, which informed subsequent national plans in these countries. In contrast, local research and advocacy over many years, supported by international experts, led to the establishment of regulatory approaches for salt reduction in Argentina and South Africa.

### Reliable local data

Lack of comprehensive, robust data to inform and monitor implementation was identified as a key barrier by all four countries. Lack of funding and/or technical capacity were the key reasons such data was absent. Accurate monitoring of salt intake using the gold standard 24-h urine method on a representative sample of the population is relatively resource intensive and requires good technical support^(46)^. Of the four countries, only Mongolia had a national measure of salt intake based on this method at baseline. Argentina and South Africa conducted regional surveys to measure salt intake using 24-h urines, while Vietnam had a national survey but used spot urines. Estimates were also different depending on the methods used, meaning there is a lack of agreement on the real baseline value. This finding is in line with other recent research showing only 13 countries conducted national surveys to measure population salt intake using 24-h urine between 2011 and 2018^(47)^ reflecting the challenges, particularly for LMIC. Further support needs to be provided to support LMIC to obtain robust baseline measures of population salt intake to ensure they are well-positioned to develop, implement and evaluate effective interventions.

Reliable data on sources of salt in the diet and salt levels in foods is also fundamental for effective salt reduction programs but was lacking in the countries in this research. Understanding the key sources of salt in diets is fundamental to identifying where most impact could be made and to inform the setting of targets to reduce salt in foods and meals^(48)^, but effectiveness is reliant on comprehensive repeat 24-h dietary recall surveys supported by up to date locally context-specific food composition tables containing the sodium content of common foods and meals. Very few LMIC have this data^(48,49)^. Future research could be directed towards developing a more effective way of rapidly assessing diets, including taking account of regional differences, with a view to identifying where to target salt reduction programs. Likewise, the establishment of comprehensive databases of processed foods, backed by laboratory analysis to assess compliance with labelling requirements, is fundamental for effective monitoring^(50)^.

### Multi-sectoral collaboration

Effective multi-sectoral collaboration was cited as a key challenge, often exacerbated by frequent changes in government or key personnel. Whilst national plans for salt reduction tend to be co-ordinated by health departments, the interventions cut across the remits of a range of departments (e.g. finance, agriculture and education) and so multi-sectoral collaboration is essential. However, despite a range of initiatives to support multi-sectoral collaboration^(51)^, countries still highlight it as a barrier to effective implementation of programs. Different ministries have their own remits, and other ministries may be less likely to contribute to agendas that are seen predominantly as a health issue^(52)^. This is relevant for most areas of NCD prevention, not just salt reduction and working in silos remains the norm for most countries regardless of income and resource level.

### Government leadership and regulatory processes

Whilst all relevant sectors need to be involved, strong government leadership is needed to prioritise and drive multi-sectoral action on salt reduction. This includes identifying and allocating appropriate funds and requesting technical support from the WHO or other relevant organisations. Effective government leadership is important in establishing policies or regulations. Governments in Argentina and South Africa legislated on limits for salt levels in foods in contrast to Mongolia and Vietnam which were planning voluntary approaches. The question of whether or not to legislate for targets (or standards) has been the subject of much discussion globally^(26,53,54)^, with some experts claiming voluntary programs have less impact^(55,56)^. The UK’s salt reduction program is often cited as one of the most successful in the world^(14,17)^. It was based on agreements with the food industry to achieve progressively more challenging targets^(57,58)^. Whilst these targets were voluntary, strong government leadership and transparent independent monitoring, as well as the threat of legislation if food companies did not make satisfactory progress towards the targets, were likely key to early success. In other high-income countries, including Australia, where voluntary processes do not seem to be working, advocates have argued that legislation for maximum levels is likely more effective^(59,60)^. Regardless, strong government leadership and robust, independent, transparent mechanisms for monitoring are key to success^(26)^. In LMIC, where there is less capacity and fewer resources for monitoring, legislation is likely a more effective and sustainable option, but monitoring and enforcement will likely remain challenging as will the large informal unregulated sector in some LMIC. Regional and global tools to support countries to establish targets^(61,62)^ and regional and global targets will be useful to support countries to take further action in this area. Whilst it is likely that these results are applicable to other nutrients that may be subject to reformulation strategies, including sugar and saturated fat, there is currently limited evidence in relation to the effectiveness of reformulation of other nutrients^(63)^. However, narrative analysis of policy implementation suggests that strong incentives for implementation alongside effective monitoring and evaluation are key drivers of success^(64)^.

### Resources and technical support for implementation and monitoring

All four countries had or were planning selected action in relation to elements of the WHO SHAKE Package. As most effective salt reduction interventions are multi-pronged^(15)^, it is important that there are adequate resources for implementing the different strands of the program, as well as co-ordination and monitoring. As well as supporting recommendations for regulation on salt targets^(26)^, global experts support the need for stronger regulation of labelling^(65)^. At the time of this research, none of the countries had mandatory Front of Pack Labelling labelling although several are now discussing its implementation. Likewise, no country had a comprehensive national program to change behaviour (although Vietnam has since launched one). This is perhaps not surprising—behaviour change programs are expensive and difficult to sustain^(66)^ and all countries highlighted a lack of funding for programs as a barrier to implementation as well as an absence of a strategic program or behaviour change framework in line with previous research^(66)^. Stakeholders in these countries reinforced the need for additional tools and resources and further technical assistance from experts not connected to industry groups with vested interests, to support effective strategic communication.

Most countries had some guidelines for either one or more settings (e.g. schools and hospitals) but not across all settings. To optimise reach and efficiency, governments should consider nutrition standards in public food procurement policies. Such policies specify that all food purchased by government or served/sold in government settings are required to adhere to a specified set of healthy nutrition standards, including but not limited to standards for sodium^(67,68)^. Introducing public procurement policies that cover all institutional settings would likely be a more sustainable scalable strategy with a broader reach than implementing different programs in different settings^(69)^.

Low sodium salts, or salty condiments such as soy sauce or fish sauce, have been identified as a sustainable and potentially scalable policy solution to reduce salt^(69,70)^; however, none of the countries in this review seemed ready to adopt this option.

### Strengths and weaknesses

This research was conducted in 2018 and there have been developments in some of the countries that are not captured in the results which is a weakness. Evaluations suggested that none of the countries had actually succeeded in reducing salt intake by 2018 when this research took place. The main changes since 2018 are that in South Africa, the government funded a one-off consumer campaign in March 2020, but the planned revisions of the legislation were not progressed due to COVID-19. In Vietnam, a large-scale consumer campaign was recently implemented. Vietnam is considering potential policy actions as part of the new NCD action plan but further plans to continue raising consumer awareness are not clear due to limited resources. Lastly, in Mongolia, the National Standard on requirements for mass catering service was established in 2019 and required salt menu labelling of meals sold in schools, hospitals, cafés and restaurants and other settings. These recent developments are important, but the main lessons from the research remain unchanged.

A key strength of this research is the deep dive into the perspectives of multiple stakeholders on salt reduction in four LMIC, triangulated with data from existing sources. The limited number of South Africa informants meant that the range of perspectives was not as broad as in the other countries, and there may have been some participant bias. However, we managed to include people from the key sectors and checked information with different sources. Language barriers were a potential source of bias in Argentina, Mongolia and Vietnam, but this was mitigated through simultaneous translation where necessary. Whilst the research was focused on population-level salt reduction interventions, many of the lessons identified will also be applicable to sugar and saturated fat reduction interventions.

A further strength of the research was the use of the Health Policy Triangle (applied to both desk research and interviews) as the framework for analysis. This facilitated a systematic assessment of factors influencing policy design and implementation and enabled cross-country comparison. More robust approaches to policy analysis have since been proposed which consider rights, gender and equity, which could further strengthen future analyses. Lastly, while the findings cannot be generalised to all LMIC, the identification of common themes and lessons will support research translation and policy design globally^(71)^.

## Conclusions

This retrospective analysis of design and implementation of policies in these countries has highlighted factors that are likely to inhibit or support effective salt reduction programs. Reliable local data about the main sources of salt, collaborative multi-sectoral implementation, strong government leadership, identification and allocation of adequate funds and increased independent technical support for implementation and monitoring, underpin effective programs. Where resources are limited, it is even more important to allocate them appropriately so robust local data on the sources of salt in the diet is essential to support prioritisation of interventions. LMIC are shouldering the major burden of NCD globally and salt reduction has been identified as a ‘best-buy’ intervention to reduce this burden. It is therefore vital that governments and international organisations mobilise adequate resources and invest in capacity strengthening to drive these initiatives forwards. This will support progress towards the global targets to reduce population salt intake by 30 % by 2025. Our findings can be used to inform future program implementation and support scale-up of salt reduction interventions in other LMIC.
